# Role of histone modification in the occurrence and development of osteoporosis

**DOI:** 10.3389/fendo.2022.964103

**Published:** 2022-08-26

**Authors:** Pan Sun, Tingrui Huang, Chen Huang, Yongjun Wang, Dezhi Tang

**Affiliations:** ^1^ Longhua Hospital, Shanghai University of Traditional Chinese Medicine, Shanghai, China; ^2^ Institute of Spine, Shanghai University of Traditional Chinese Medicine, Shanghai, China; ^3^ Shanghai University of Traditional Chinese Medicine, Shanghai, China

**Keywords:** osteoporosis, histone modification, osteoblast, osteoclast, differentiation

## Abstract

Osteoporosis is a systemic degenerative bone disease characterized by low bone mass and damage to bone microarchitecture, which increases bone fragility and susceptibility to fracture. The risk of osteoporosis increases with age; with the aging of the global population, osteoporosis is becoming more prevalent, adding to the societal healthcare burden. Histone modifications such as methylation, acetylation, ubiquitination, and ADP-ribosylation are closely related to the occurrence and development of osteoporosis. This article reviews recent studies on the role of histone modifications in osteoporosis. The existing evidence indicates that therapeutic targeting of these modifications to promote osteogenic differentiation and bone formation may be an effective treatment for this disease.

## Introduction

Osteoporosis is a common skeletal disease characterized by a decrease in bone mass, changes in bone microarchitecture, and increased bone fragility and risk of fracture. Pain, fractures, and other complications of osteoporosis are associated with high rates of death and disability. Bone homeostasis (1), which is maintained under physiologic conditions by a balance between bone formation and resorption during bone remodeling, is critical for ensuring the long-term stability of bone morphology and strength (2). Although the pathogenesis of osteoporosis is not fully understood, an imbalance in bone homeostasis during bone reconstruction whereby bone resorption exceeds bone formation is a major cause (1). The function of osteoblasts and osteoclasts is regulated and influenced by many factors (3–5), and epigenetic studies have provided evidence for the role of histone modifications in the development of osteoporosis (6–8).

Histones H1, H2A, H2B, H3, and H4 are small proteins enriched in positively charged basic amino acids (arginine [R] and lysine [K]) that interact with the negatively charged phosphate groups in DNA and are enveloped by DNA to form nucleosomes, the basic structural unit of chromatin. Histone N-terminal R and K undergo covalent post-transcriptional modifications such as methylation, acetylation, ubiquitination, and ADP-ribosylation that affect histone binding to DNA and alter the structure and state (open *vs.* closed) of chromatin (9), and also affect the binding of transcription factors at gene promoters to influence gene regulation (10, 11). Histone modifications occur at every stage of development, growth, and aging and are a key aspect of epigenetic regulation that has been linked to the development and progression of multiple diseases (12).

There is increasing evidence that dysregulation of histone modification (methylation, acetylation, ubiquitination, and ADP-ribosylation) and impaired function of related enzymes contribute to the development of osteoporosis. However, molecular-level details of the relationship between these modifications and disease pathogenesis are lacking, and the full clinical significance of histone modifications in osteoporosis remains to be determined (13, 14). Nonetheless, histone modifications may be important for the diagnosis, treatment, and prognosis of osteoporosis and are potential therapeutic targets (15).

In this review, we summarize the current state of knowledge on histone modifications in osteoporosis. Many studies have demonstrated that regulators of histone modifications and their targets function in a complex regulatory network in cells. We discuss the evidence for targeting the regulation of histone modifications as a treatment for osteoporosis, as well as the potential utility of these modifications as disease markers.

## Histone methylation

Histone methylation usually occurs at R and K residues at the N terminus of histones. K residues can be mono-, di- or trimethylated and R residues can be mono- or dimethylated. Histone methylation positively and negatively regulates gene expression: H3K4me1, H3K4me3, H3K36me3, and H3K79me2 are associated with the activation of gene transcription whereas H3K27me3 and H3K9me3 are associated with transcriptional repression (16). Histone methylation is regulated by histone methyltransferases (HMTs) and histone demethylases (HMDs) (17). Thus, the expression of genes related to bone homeostasis and osteoporosis can be regulated by altering the level of histone methylation (**Table 1**).

**Table 1:** Histone methyltransferases, histone demethylases, target histone sites, and their roles in the occurrence and development of osteoporosis.

HMTs and HMDs	Target histone sites	Target genes	Function
PRMT1/PRMT4 (CARM1)	H4R3me2a, H3R17me2a	*CYP24A1*	Activates *CYP24A1* gene in osteoblasts (18).
PRMT5	H4R3me2s (19), H3R8me2s (20)	*ISG (* 19), *CXCL10* and *RSAD2* (20)	Regulates osteogenic differentiation of BMSCs (19). Inhibits PRMT5 from suppressing osteoclast differentiation (20).
PADI4		*Runx2*	Promotes osteoblast mineralization (21).
KMT1A (Suv39h1)	H3K9me2/3	*Runx2*	Delays osteoblast differentiation (22).
KMT1C (G9a, EHMT2)	H3K9me2 (23), H3K27me1 (24)	Runx2 (23), MMP-9 (24)	Regulates proliferation and differentiation of cranial bone cells (23) Induces expression of osteoclastogenesis-related genes and promotes osteoclast differentiation (24).
KMT1D (EHMT1)	H3K9me2	*Runx2*	Suppresses osteogenic differentiation of mesenchymal stem cells (25).
KMT1E (ESET,SETDB1)	H3K9me3		Regulates osteoblast differentiation of MSCs (26).
KMT6 (EZH2)	H3K27me3	*Wnt4*, *Foxc1*	Enhances both osteogenesis and osteoclastogenesis (27).
Mll-COMPASS complexes	H3K4me3	*Runx2*, *p57*	Promotes *Runx2*, *p57* gene transcription (28).
KMT2D (MLL4)	H3K4me1	*Runx2*	Promotes osteoblast differentiation (29).
KMT4 (DOT1L)	H3K9me2	*CD9*, *MMP-9*	Inhibits osteoclastogenesis and protects against osteoporosis (30).
KDM1 (LSD1)	H3K4me1 (31), H3K4me2 (29)	*Runx2* (31), *Wnt7b*, *BMP2* (29)	Inhibits osteoblast differentiation of C2C12 cells (31). Inhibits osteogenic differentiation of BMSCs (29).
KDM4A (JMJD2A)	H3K9me3	*Sfrp4*, *C/ebpα*	Promotes adipogenic differentiation and inhibits osteogenic differentiation (32).
KDM4B (JMJD2B)	H3K9me3	*Runx2*, *Ccnd1*	Promotes osteogenic differentiation of BMSCs and maintains bone–fat balance (33, 34).
KDM5A (JARID1A, RBP2)	H3K4me3	*Runx2*	Inhibits BMP2-induced osteogenesis of MSCs (35). Inhibits osteogenic differentiation of human adipose-derived stromal cells (36).
KDM5B (JARID1B, PLU-1)	H3K4me3	*Runx2*	Enhances osteoblast differentiation (37).
KDM6A (UTX)	H3K27me3		Inhibits adipogenic differentiation and promotes osteogenic differentiation of BMSCs (38).
KDM6B (JMJD3)	H3K27me3	*Runx2*, *Osx* (39), *NFATc1* (40)	Regulates osteoblast differentiation (39).Promotes osteoclast differentiation (40).

HDM, histone demethylase; HMT, histone methyltransferase.

PRMT, Protein arginine methyltransferase; CARM1, Coactivator associated arginine methyltransferase 1; CYP24A1, Cytochrome P450 family 24 subfamily A member 1; ISG, Interferon-stimulated gene; CXCL10, C-X-C motif chemokine ligand 10; RSAD2, Radical S-adenosyl methionine domain containing 2; PADI4, Peptidyl arginine deiminase 4; Runx2, Runt-related transcription factor 2; Suv39h1, Suppressor of variegation 3-9 homolog 1; KMT, Calmodulin-Lysine N-methyltransferase; EHMT, Euchromatic histone lysine methyltransferase; MMP, Matrix metallopeptidase; ESET,SETDB1, SET domain bifurcated histone lysine methyltransferase; EZH2, Enhancer of zeste 2 polycomb Repressive Complex 2 Subunit; Foxc1, Forkhead box C1; DOT1L, DOT1 Like Histone Lysine Methyltransferase; KDM, LSD, Lysine demethylase; BMP2, Bone morphogenetic protein 2; JMJD, JmjC domain-containing histone demethylation protein; Sfrp4, Secreted frizzled related protein 4; C/ebpα, CCAAT enhancer binding protein alpha; Ccnd1, Cyclin D1; RBP2, Retinol binding protein 2; UTX, Utx histone demethylase; Osx, Osterix; NFATc1, Nuclear factor of activated T cells 1.

Several genes are regulated by HMTs or HMDs during the differentiation and maturation of osteoblasts and osteoclasts (6, 14, 15). Suv39h1 is an H3K9 methyltransferase that can modify H3K9 with two or three methyl groups. H3K9me2 and H3K9me3 bind to the promoter of *Runx2 (* 22)—a key gene involved in osteoblast differentiation and maturation (41)—and suppress gene transcription, thereby delaying osteoblast differentiation, which may be relevant to the pathogenesis of osteoporosis. EZH2 is a trimethyltransferase of H3K27; H3K27me3 activates transcription of the *Wnt4* gene in osteoblasts to promote osteogenic differentiation (27). H3K27me3 also activates the transcription of *Foxc1* in osteoclasts and promotes osteoclast differentiation (27). Thus, histone methylation plays opposing regulatory roles in the pathogenesis of osteoporosis. However, some of the evidence regarding the function of histone methylation in bone homeostasis is controversial. For instance, JMJD2A and JMJD2B are H3K9me3 demethylases; in one study, demethylation of H3K9me3 by JMJD2A promoted adipogenic differentiation and inhibited osteogenic differentiation (32), whereas another study found that H3K9me3 demethylation by JMJD2B promoted osteogenic differentiation of bone marrow-derived stem cells (BMSCs) and maintained bone–fat balance (33).

## Histone acetylation

### Histone acetyltransferases and histone deacetylases

Acetylation was one of the first histone modifications found to affect transcriptional regulation and is therefore the most widely studied. Acetylation causes K residues in the N-terminal histone tails protruding from nucleosomes to become negatively charged, which repels negatively charged DNA and leads to relaxation of the chromatin structure. The open chromatin conformation allows transcription factors to bind more easily, resulting in an increase in gene expression (42, 43). Thus, histone acetylation is mainly associated with gene activation; it is known to be involved in cell cycle regulation, cell proliferation, and apoptosis, cellular differentiation, DNA replication and repair, nuclear import, and neuronal inhibition (44, 45), whereas dysregulation of histone acetylation has been implicated in osteoporosis progression (46–48).

Histone acetylation is regulated by HATs and HDACs (**Table 2**). The HAT family includes GNAT (HAT1, GCN5, PCAF) and MYST (Tip60, MOF, MOZ, MORF, HBO1) as well as CREB-binding protein (CBP)/p300, which share very high sequence similarity in the bromodomain, cysteine-histidine-rich region, and HAT structural domain and specifically bind phosphorylated CREB to enhance its transcription of cAMP-responsive genes.

**Table 2:** Histone acetyltransferases, histone deacetylases, and their roles in the occurrence and development of osteoporosis.

HATs and HDACs	Target genes	Function
KAT2A (GCN5)	*Wnt*, *NF-κB*	Enhances osteogenic differentiation ability of BMSCs (49–51).
KAT2B (PCAF)	*BMP*, *Runx2* (52), *CXCL12* (53) NFATc1 (54)	Promotes osteogenic differentiation of MSCs (52, 53). Promotes osteoclast differentiation (54)
CBP/p300	*Runx2* (55), *NFATc1* (54)	Promotes osteoblast differentiation (55). Promotes osteoclast differentiation (54).
HDAC1	*IGF-1*	Prevents achievement of peak bone mass by inhibiting IGF-1 expression in the liver and IGF-1 signaling in bone (56).
HDAC2	*SP7* (57), *AKT*, *FoxO1* (58)	Inhibits osteogenic differentiation of MSCs (57). Activates Akt and thereby suppresses FoxO1 transcription, resulting in enhanced osteoclastogenesis (58).
HDAC3	*NF-κB*	Controls bone remodeling by suppressing the responsiveness of osteoclast lineage cell to RANKL (59).
HDAC4	*MEF2C*, *MMP13* (60), *Runx2* (61)	HDAC4 interacts with MEF2C at the *MMP13* promoter and inhibits *MMP13* gene transcription (60). Deacetylates and degrades Runx2, leading to reduced osteoblast function (61).
HDAC5	*MEF2C* (62), *NFATc1* (54)	HDAC5 binds and inhibits the function of MEF2C and decreases SOST expression in osteocytes (62). Reduces RANKL- or PCAF-mediated NFATc1 acetylation, stability, and transactivation activity and suppresses osteoclast differentiation (54)
HDAC6	*Runx2*	Inactivates *Runx2* promoter to block osteogenesis of BMSCs (48).
HDAC7	*Mitf*	Represses Mitf function and inhibits osteoclast differentiation (63). Represses Runx2 expression and suppresses osteoblast maturation (64).
HDAC8	*Runx2*	Suppresses osteogenic differentiation of BMSCs by inhibiting H3K9ac and Runx2 activity (65).
HDAC11	*11β-HSD2*	Suppresses osteogenic differentiation of BMSCs by downregulating H3K9ac and 11β-HSD2 expression (66).

HAT, histone acetyltransferases; HDAC, histone deacetylase.

KAT2A(GCN5), Lysine acetyltransferase 2A; NF-κB, Nuclear factor kappa B; KAT2B(PCAF), Lysine acetyltransferase 2B; CXCL12, C-X-C motif chemokine ligand 12; CBP, CREB binding protein, IGF-1, Insulin like growth factor 1; SP7, Sp7 transcription factor; Akt, AKT serine/threonine kinase; RANKL, NF-κB ligand-receptor activator; MEF2C, Myocyte enhancer factor 2C; SOST, Sclerostin; Mitf, Melanocyte inducing transcription factor; 11β-HSD2, Hydroxysteroid 11-beta dehydrogenase 2.

CBP/p300 has a regulatory role in bone formation, targeting transcription factors such as *Runx2* during osteoblast differentiation. During parathyroid hormone-induced osteoblast differentiation, phosphorylated HDAC4 dissociates from Runx2, which interacts with CBP/P300 (67). Transforming growth factor beta-1(TGF-β1) and BMP2 stimulate ERK-mediated phosphorylation of Runx2 to promote its interaction with CBP/p300 (68). BMP2 activates SMAD1/5, leading to CBP/p300-mediated acetylation of *Runx2*, which enhances the expression of osteogenic genes such as alkaline phosphatase(*ALP*) and collagen type I (*COL-I*) (69, 70).

*Runx2* (41), *Sp7* (71), and *FoxO1* (72) are important transcription factors for osteoblast differentiation and maturation that induce the transcription of downstream osteogenesis-related genes such as *ALP*, osteocalcin(*OCN*), osteopontin(*OPN*), and *COL-1* and promote the maturation and mineralization of osteoblasts. As *Runx2* transcription is initiated, PCAF and CBP/p300 acetylate histone H3 to promote osteoblast differentiation and maturation (52, 55). However, the transcription of *Runx2*, *SP7*, and *FoxO1* was shown to be inhibited after HDAC reduced the histone acetylation level, leading to suppression of osteoblast differentiation and maturation (48, 57, 58, 61, 65) (**Table 2**). The differentiation and maturation of osteoclasts are controlled by the transcription factors *NFATc1* (73) and *NF-κB* (74), among others. PCAF and CBP/p300 acetylate histone H3 to promote osteoclast differentiation, whereas differentiation is inhibited by HDAC-mediated H3 deacetylation (52, 55) (**Table 2**). These findings suggest that the balance between the activities of HATs and HDACs is critical for the regulation of transcription factors involved in osteoblast and osteoclast differentiation.

### Sirtuins

**Figure 1:**
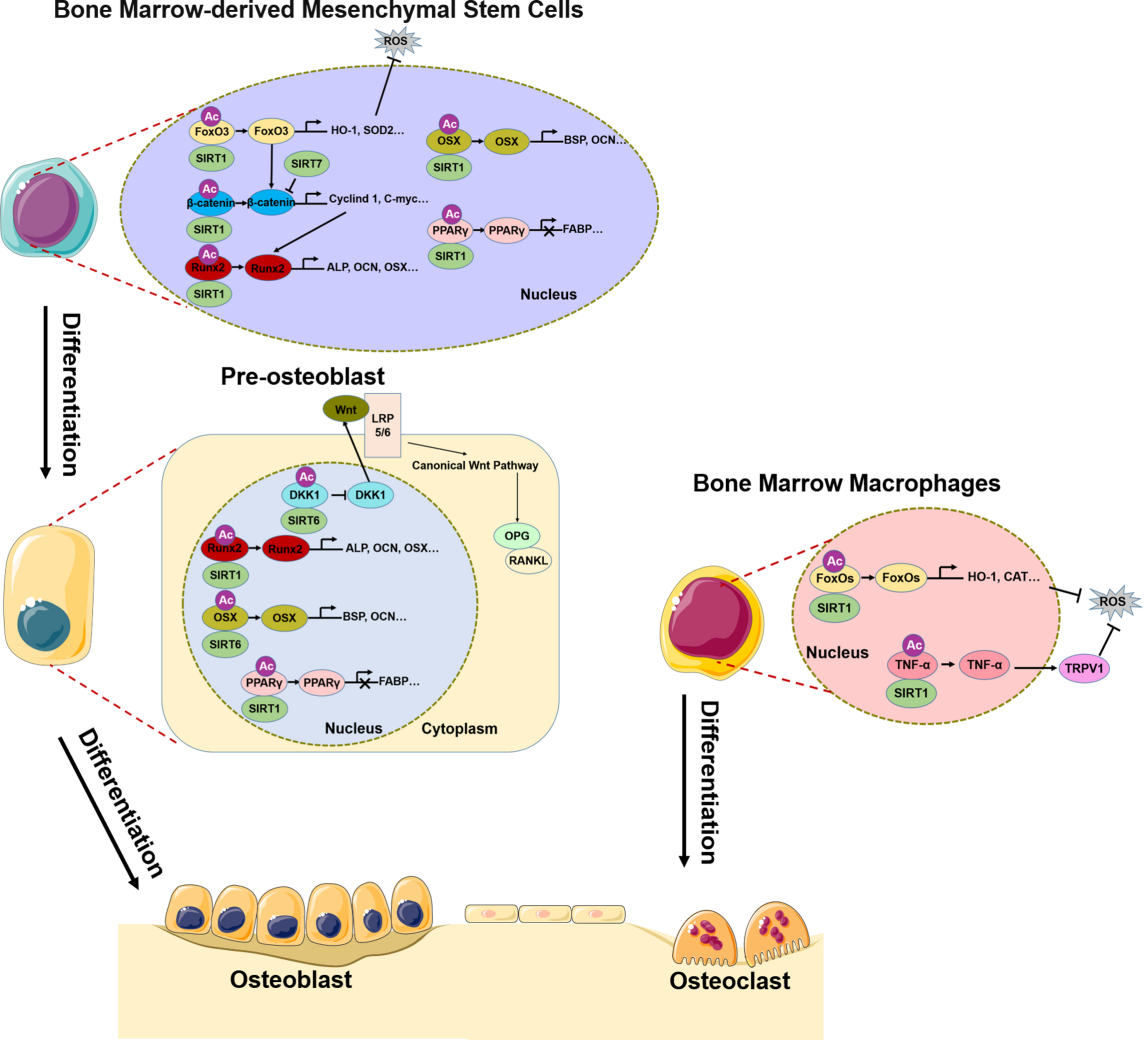
Role of SIRT1/6/7 in bone remodeling. In BMSCs, SIRT1, SIRT6, and SIRT7 promote osteogenic differentiation by regulating transcription factors *FoxO3*, *β-catenin*, *Runx2*, *Osx*, and *PPARγ*. In pre-osteoblasts, SIRT6 not only regulates *Runx2* and *Osx* transcription, but also inhibits *DKK1* transcription, activates canonical Wnt signaling, and promotes osteogenic differentiation. In BMMs, SIRT1 inhibits osteoclast differentiation by regulating *FoxO* and *TNF-α* transcription, which results in ROS scavenging.

SIRTs are highly conserved HATs that transfer the acetyl group of a substrate to the ADP-ribosyl moiety of nicotinamide adenine dinucleotide (NAD+), NAD+ dependent protein deacetylation consumes NAD+, transfers the acetyl group from the lysine to ADP-ribose to form 2’-O-acetyl-ADPR, nicotinamide, and a deacetylated lysine. The SIRT family comprises SIRT1–7, of which SIRT1, SIRT6, and SIRT7 are mainly localized in the nucleus (75, 76). It should be noted that, most of the discussed Sirtuin effects do not exhibit their function by direct deacetylation of histones, but by deacetylation of other targets. SIRT1 deacetylates H3K9 and regulates of a variety of physiologic processes including metabolism, immune response, and aging (77), and has been linked to osteoporosis (78). The Wnt/β-catenin signaling pathway plays a central role in the differentiation of BMSCs into osteoblasts (79–82); SIRT1 deacetylates K49R or K345R of β-catenin, promoting its entry into the nucleus where it induces the transcription of osteogenic differentiation-related genes such as *Cyclin D1* and *C-myc* and promotes the expression of *Runx2* (83). SIRT1 also directly deacetylates *Runx2*, which in turn induces the transcription of genes that promote osteogenic differentiation of BMSCs (84). peroxisome proliferators-activated receptors gamma(*PPARγ*) is an important transcription factor for the adipogenic differentiation of BMSCs (85), which inhibits osteogenic differentiation and disrupts bone–fat balance; this is restored by reduction of *PPARγ* acetylation level by SIRT1 and consequent suppression of the adipogenic differentiation of BMSCs (86, 87) (**Figure 1**). In pre-oisteoblasts, SIRT1 also down-regulates PPARγ to promote osteogenic differentiation (88). Excessive reactive oxygen species (ROS) production in BMSCs under oxidative stress affects osteogenic differentiation (72). SIRT1 reduces the acetylation level of *FoxO3*, a transcription factor involved in the cellular response to oxidative stress, leading to *FoxO3* transcription and the expression of antioxidant enzymes such as heme oxygenase 1(*HO-1*) and superoxide dismutase 2(*SOD2*) (89, 90). The subsequent removal of excess ROS in BMSCs restores their osteogenic differentiation capacity. *FoxO3* transcription also promotes the expression of β-catenin, resulting in osteogenic differentiation (90) (**Figure 1**). Bone resorption depends on ROS produced in osteoclasts; SIRT1 was shown to reduce FoxO acetylation level in bone marrow macrophages(BMMs) and promote the expression of antioxidant enzymes that clear ROS (91). At the same time, SIRT1 reduced *TNF-α* acetylation level, thereby increasing the expression of transient receptor potential cation channel subfamily V member 1(*TRPV1*), ROS scavenging, and inhibiting bone resorption (92) (**Figure 1**).

SIRT6 and SIRT7 are involved in chromatin regulation and play multiple roles in metabolism, aging, and disease. In osteoblasts, SIRT6 reduces H3K9 acetylation level and promotes the transcription of osteogenic transcription factors *Runx2* and *Osx* and the expression of osteogenic genes (93). Additionally, SIRT6 negatively regulates the expression of dickkopf1 (*DKK1*) (93), a secreted protein that binds to the Wnt receptor LRP5/6; this induces rapid endocytosis and reduces LRP5/6 in the cell membrane (94), thereby blocking the canonical Wnt signaling cascade. Blocking the Wnt pathway leads to a reduction in the synthesis of osteoprotegerin (OPG), which competitively binds to RANKL to inhibit osteoblast differentiation (95). Thus, reducing *DKK1* expression facilitates the nuclear entry of β-catenin, which initiates the transcription of target genes that promote osteoblast differentiation while inhibiting those involved in osteoblast differentiation. Additionally, SIRT7 has the effect of reducing β-catenin level in BMSCs (96), although the significance of this observation in the context of bone homeostasis and osteoporosis remains unclear.

## Histone ubiquitination

All histones can be ubiquitinated, with H2A and H2B being the most frequent targets. Histone ubiquitination plays a central role in the DNA damage response. Monoubiquitination of H2A, H2B, and H2AX has been observed at DNA double-strand break sites; the most common forms are monoubiquitination of K119 on H2A and K123 (yeast)/K120 (vertebrate) on H2B. H2A and H2B monoubiquitination was shown to be associated with gene silencing and transcriptional activation, respectively (97, 98).

Ring finger protein 40 (RNF40), an E3 ubiquitin ligase that monoubiquitinates H2B (99), was found to regulate the transcription of the osteogenic genes bone gamma-carboxyglutamate protein (*BGLAP*), *ALP*, and glucose-6-phosphate dehydrogenase (*G6PD*) to induce osteogenic differentiation of human BMSCs (100). The *Tnfsf11* gene (encoding RANKL) is a target gene of H2Bub1 (101). It was reported that H2Bub1, whose expression was induced by RNF40, was required in the early stages of osteoblast differentiation and modulated osteoblast function by regulating VDR-induced *Tnfsf11* expression in crosstalk between osteoblasts. The long noncoding RNA ODIR1 was significantly downregulated during osteogenic differentiation of human umbilical cord mesenchymal stem cells (hUC-MSCs); the interaction of ODIR1 with F-box protein 25 (*FBXO25*) increased monoubiquitination of H2BK120 (H2BK120ub), promoted the trimethylation of H3K4 (H3K4me3), and induced the expression of the transcription factor Osx, thereby enhancing the expression of the osteoblast markers *OCN*, *OPN*, and *ALP*. Thus, ODIR1 negatively regulates the osteogenic differentiation of hUC-MSCs *via* the FBXO25/H2BK120ub/H3K4me3/Osx axis (102).

Myb like, SWIRM and MPN domains 1(Mysm1) is an H2A deubiquitinating enzyme that regulates osteoblast differentiation and maturation by promoting *Runx2* expression in osteoblasts. *Mysm1^−/−^* mice exhibit significant skeletal deformation and osteoporosis; however, osteogenic differentiation capacity was not significantly affected in MSCs lacking Mysm1. In *p53^−/−^Mysm1^−/−^* double knockout mice, p53 deletion rescued the skeletal defects and bone loss caused by *Mysm1* deficiency. On the other hand, loss of p53 did not restore *Runx2* expression in *Mysm1^−/−^* osteoblasts although MSCs proliferation and osteogenic differentiation was enhanced (103).

## Histone ADP-ribosylation

Poly (ADP-Ribose) polymerases (PARPs) (also known as ARTDs), are a family of 17 proteins, some of PARPs are mono-ADP-ribosyl transferases and some poly. PARPs uses NAD+ as substrate to transfer single or straight or branched ADP-ribose to itself or other target proteins, thus regulating various cellular responses. The most widely studied member of the PARPs family is PARP1, which is thought to play a role in DNA repair (104). PARP1 binds to DNA damage sites and catalyzes its own ADP-ribosylation reactions and the trans-modification of local substrate proteins, including DNA repair proteins, histones and other chromatin-associated proteins, to promote the repair of DNA lesions, influence chromatin structure and gene transcription (105, 106).

Induction of NFATc1 by macrophage colony-stimulating factor (M-CSF) and RANKL is essential for macrophage differentiation into osteoblasts (73). ADP-ribosylation of H2B at serine 7 by PARP1 reduced the occupancy of this histone at the *NFATc1* promoter, reducing NFATc1 expression and osteoclast formation (107). Moreover, PARP1 inhibited the expression of osteoclast-promoting genes *via* regulation of histone ADP-ribosylation at the *IL-1β* promoter, which increased *IL-1β* expression (108). M-CSF induced PARP1 self–ADP-ribosylation in macrophages, resulting in PARP1 cleavage at D214 and its subsequent degradation; this stimulated RANKL-induced osteoclast differentiation and osteoclast maturation, whereas osteoclastogenesis was inhibited by expression of the cleavage-resistant D214N mutant form of PARP1 (109). The PARP inhibitor olaparib decreased ALP activity in preosteoblastic MC3T3-E1 cells and inhibited the formation of the mineralized nodules that characterize osteoblasts (110). In contrast, inhibiting the enzymatic activity of poly(ADP-Ribose) glycohydrolase (PARG) using the inhibitor PDD00017273 enhanced Runx2 PARylation (ribosylation) and osteoblast formation while having no effect on *PARG* mRNA expression.

## Summary and prospects

Osteoporosis is a complex disease whose pathogenesis remains unclear, although an imbalance in bone homeostasis during bone reconstruction is thought to contribute. Accumulating evidence indicates that histone modifications play an essential role in various diseases including osteoporosis. Specifically, changes in the levels of histone modification and related enzymes can lead to altered expression of genes involved in bone formation or bone resorption, resulting in an imbalance in bone homeostasis and abnormal bone reconstruction.

According to this review, it can be proved that histone modifications play important role in osteoporosis, and related research results have been applied to the diagnosis and treatment of osteoporosis. Identifying specific biomarkers associated with osteoporosis will significantly improve the clinical diagnosis and treatment of this disease. In recent years, drugs targeting the activity of histone-modifying enzymes have been evaluated in studies focused on osteoporosis treatment. As an example, SOST—which negatively regulates bone formation—is regulated by a class I HDAC (111); meanwhile, the class I HDAC inhibitor MS-275 was shown to promote bone formation (112).

There have been a limited number of studies on the effects of histone modification on bone homeostasis, leaving many open questions. For example, the relationship between abnormal regulation of histone modifications in genes related to bone homeostasis and the development of osteoporosis requires clarification. It is unclear whether histone modifications are a primary cause of osteoporosis or a secondary effect, although it is clear that the effects of histone modifications are associated with the pathogenesis of osteoporosis. More in-depth mechanistic studies are expected to provide a better understanding of the effect of histone modification on individual bone cell type by using tissue-specific deletion and transgenic animal models. Further studies in this area can provide insight not only into disease pathogenesis, but also novel diagnostic biomarkers and therapeutic targets.

## Author contributions

PS and TH performed the literature review and wrote the manuscript. CH consulted and summarized the literature. YW provided supervision and advisory during the writing process. DT reviewed the literature and edited the manuscript. All authors contributed to the article and approved the submitted version.

## Funding

This work was supported in part by the National Natural Science Foundation of China (81730107, 81973883), the Innovation Team and Talents Cultivation Program of National Administration of Traditional Chinese Medicine (ZYYCXTD-C-202202), the National Key R&D Program of China (2018YFC1704300), the Shanghai Scientific Research Project (19ZR1458000), the Shanghai Traditional Chinese Medicine Medical Center of Chronic Disease (2017ZZ01010), and the Innovation Team of the Ministry of Education (IRT1270).

## Conflict of interest

The authors declare that the research was conducted in the absence of any commercial or financial relationships that could be construed as a potential conflict of interest.

## Publisher’s note

All claims expressed in this article are solely those of the authors and do not necessarily represent those of their affiliated organizations, or those of the publisher, the editors and the reviewers. Any product that may be evaluated in this article, or claim that may be made by its manufacturer, is not guaranteed or endorsed by the publisher.
